# Supplementary figures and images for: The transcription factor Bach2 negatively regulates murine natural killer cell maturation and function
Source: eLife. 2022 Oct 3;11:e77294. doi: 10.7554/eLife.77294 (PMC9560152; doi:10.7554/eLife.77294)

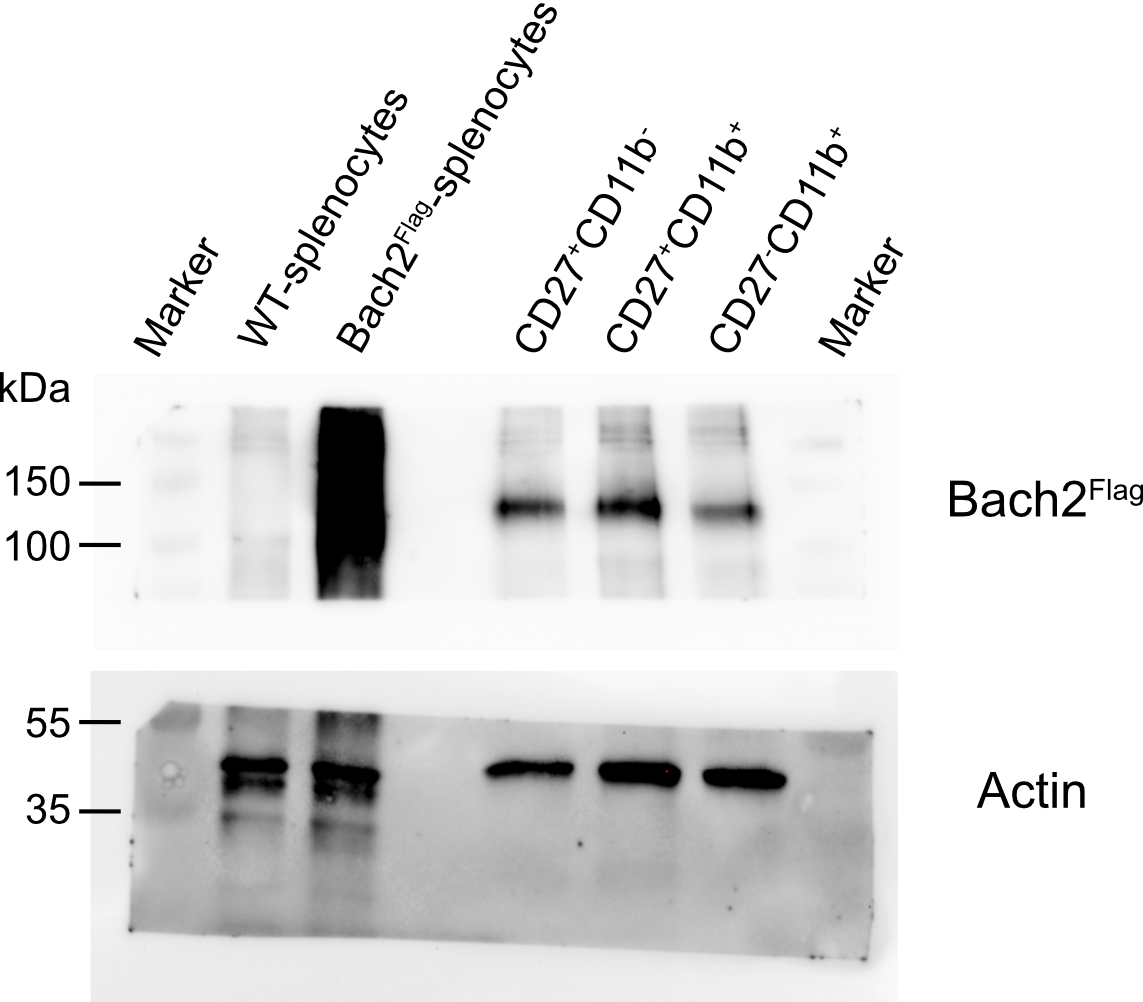

Supplement: Figure 1—source data 1. — Splenic NK cells were enriched from splenocytes of Bach2Flag mice. Enriched NK cells (CD3-NK1.1+) from Bach2Flag mice were further sorted into CD27+CD11b-, CD27+CD11b+, and CD27-CD11b+ subsets. Bach2 expression in the subsets was detected using Anti-FLAG M2-Peroxidase (HRP) antibody by western blot. Expression of Actin was used as an internal control. Splenocytes from wild-type (WT) mice were used as negative control. Splenocytes from Bach2Flag mice were used as positive control. Two individual experiments have been done with one mouse each time. [file elife-77294-fig1-data1.zip › Figure 1-source data 1/Figure 1-source data 1.tiff]

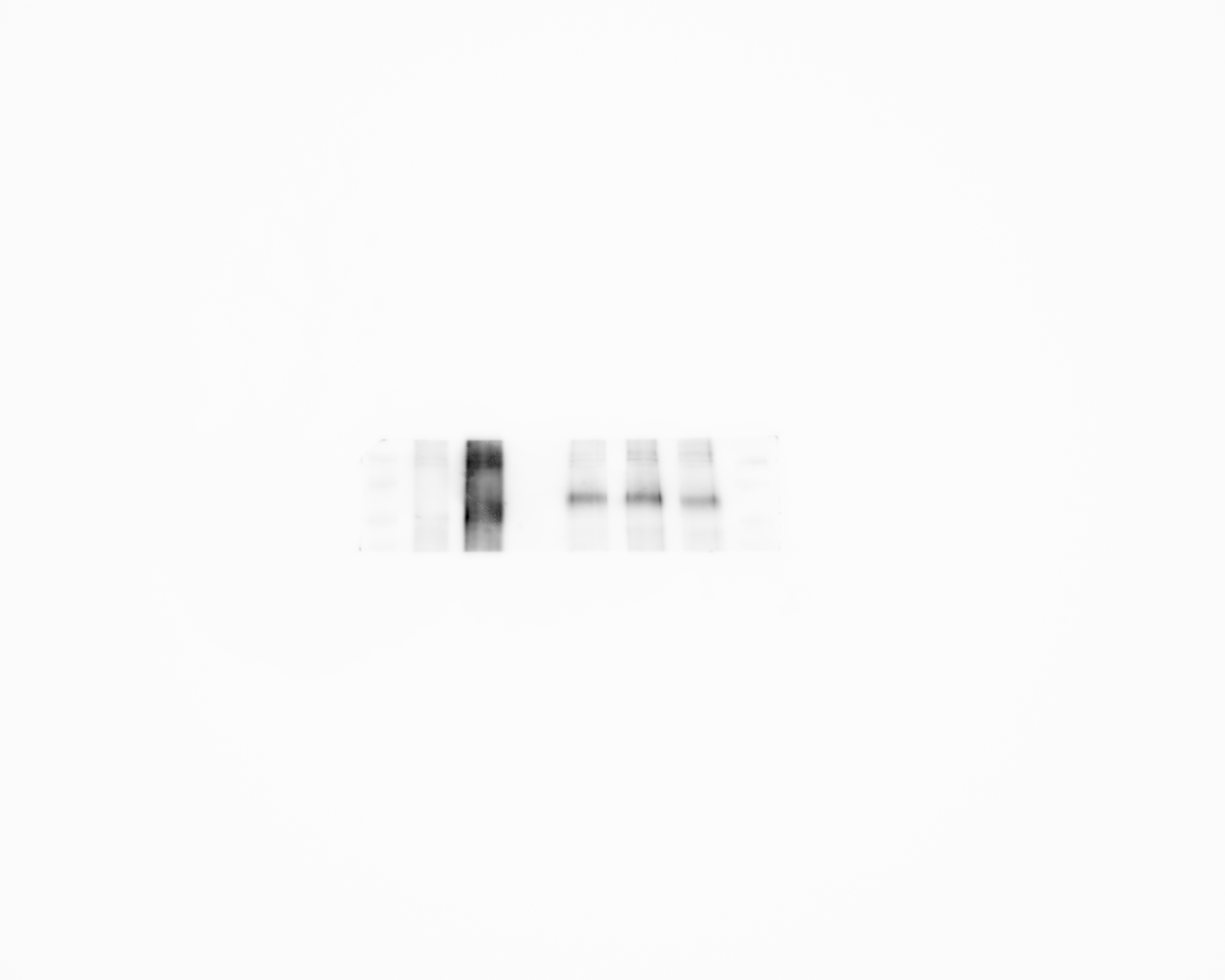

Supplement: Figure 1—source data 1. — Splenic NK cells were enriched from splenocytes of Bach2Flag mice. Enriched NK cells (CD3-NK1.1+) from Bach2Flag mice were further sorted into CD27+CD11b-, CD27+CD11b+, and CD27-CD11b+ subsets. Bach2 expression in the subsets was detected using Anti-FLAG M2-Peroxidase (HRP) antibody by western blot. Expression of Actin was used as an internal control. Splenocytes from wild-type (WT) mice were used as negative control. Splenocytes from Bach2Flag mice were used as positive control. Two individual experiments have been done with one mouse each time. [file elife-77294-fig1-data1.zip › Figure 1-source data 1/Figure 1-source data 2.tif]

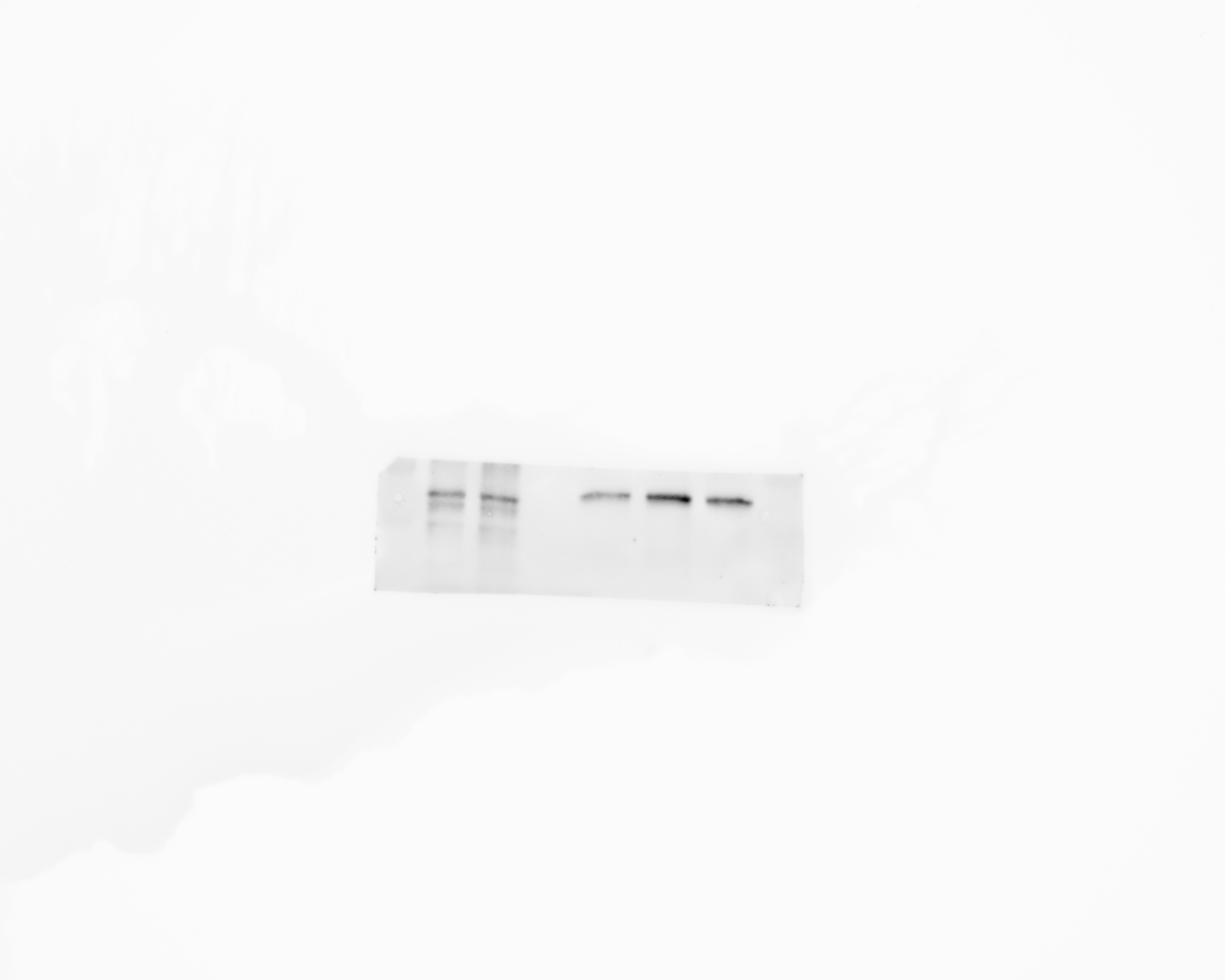

Supplement: Figure 1—source data 1. — Splenic NK cells were enriched from splenocytes of Bach2Flag mice. Enriched NK cells (CD3-NK1.1+) from Bach2Flag mice were further sorted into CD27+CD11b-, CD27+CD11b+, and CD27-CD11b+ subsets. Bach2 expression in the subsets was detected using Anti-FLAG M2-Peroxidase (HRP) antibody by western blot. Expression of Actin was used as an internal control. Splenocytes from wild-type (WT) mice were used as negative control. Splenocytes from Bach2Flag mice were used as positive control. Two individual experiments have been done with one mouse each time. [file elife-77294-fig1-data1.zip › Figure 1-source data 1/Figure 1-source data 3.tif]
